# Efficacy and Safety of S1P1 Receptor Modulator Drugs for Patients with Moderate-to-Severe Ulcerative Colitis

**DOI:** 10.3390/jcm12155014

**Published:** 2023-07-30

**Authors:** Sarah Bencardino, Ferdinando D’Amico, Ilaria Faggiani, Francesca Bernardi, Mariangela Allocca, Federica Furfaro, Tommaso Lorenzo Parigi, Alessandra Zilli, Gionata Fiorino, Laurent Peyrin-Biroulet, Silvio Danese

**Affiliations:** 1 Gastroenterology and Endoscopy, IRCCS Ospedale San Raffaele and Vita-Salute San Raffaele University, 20132 Milan, Italy; bencardino.sarah@hsr.it (S.B.); damico_ferdinando@libero.it (F.D.); faggiani.ilaria@hsr.it (I.F.); bernardi.francesca@hsr.it (F.B.); allocca.mariangela@hsr.it (M.A.); furfaro.federica@hsr.it (F.F.); parigi.tommaso@hsr.it (T.L.P.); zilli.alessandra@hsr.it (A.Z.); fiorino.gionata@hsr.it (G.F.); 2 Department of Biomedical Sciences, Humanitas University, Pieve Emanuele, 20072 Milan, Italy; 3 Department of Gastroenterology, University of Lorraine, CHRU-Nancy, F-54000 Nancy, France; peyrinbiroulet@gmail.com; 4 Department of Gastroenterology, Nancy University Hospital, F-54500 Vandœuvre-lès-Nancy, France; 5 INSERM, NGERE, University of Lorraine, F-54000 Nancy, France; 6 INFINY Institute, Nancy University Hospital, F-54500 Vandœuvre-lès-Nancy, France; 7 FHU-CURE, Nancy University Hospital, F-54500 Vandœuvre-lès-Nancy, France; 8 Groupe Hospitalier privé Ambroise Paré-Hartmann, Paris IBD Center, F-92200 Neuilly sur Seine, France; 9 Division of Gastroenterology and Hepatology, McGill University Health Centre, Montreal, QC H4A 3J1, Canada

**Keywords:** ulcerative colitis, inflammatory bowel disease, S1P1 receptor modulators, ozanimod, etrasimod, VTX-002, IBD

## Abstract

Ulcerative colitis (UC) is a chronic inflammatory bowel disease (IBD) that negatively impacts patients’ quality of life. In the last decades, the therapeutic options available for the management of patients with moderate to severe UC have increased significantly, including not only biological drugs but also small molecules. However, there is a persistent need to develop new drugs that act on new targets while minimizing the risk of adverse events. Sphingosine-1-phosphate (S1P) is a membrane-derived lysophospholipid. The S1P gradient between tissues and the circulatory system has a key role in regulating the trafficking of immune cells as autoreactive B and T lymphocytes. S1P receptor modulators could be a safe and efficacious alternative mechanism for reducing inflammation in immune-mediated disorders, including UC, by reducing lymphocyte egress from the lymph nodes to the bloodstream. Several S1P receptor modulators have been developed and tested in UC. Ozanimod is already approved by Food and Drug Administration (FDA) and European Medical Agency (EMA), while etrasimod and VTX002 are still under approval. Oral administration route, rapidity and reliable safety profile are the main advantages of this class of drugs. The aim of this review is to summarize the available evidence for the efficacy, safety, and pharmacokinetics of ozanimod, etrasimod, and VTX002 in UC.

## 1. Introduction

Ulcerative colitis (UC) is a chronic inflammatory disease characterized by alternating phases of remission and recurrence [1]. It is associated with decreased health-related quality of life and significant financial burden [2,3]. This disease is more frequent in North America and Western Europe, but the incidence is also increasing in less industrialized countries. The overall incidence is 1.2 to 20.3 cases per 100,000 persons per year, and the prevalence is 7.6 to 245 cases per 100,000 per year [4,5].

Tumor necrosis factor α inhibitors (infliximab, adalimumab, and golimumab) revolutionized UC treatment nearly 20 years ago, allowing for better disease control by raising rates of clinical remission, endoscopic remission, and corticosteroid-free remission, as well as improving patients’ quality of life [6,7,8].

Vedolizumab and, more recently, ustekinumab, biologics with different targets, were later authorized for the treatment of moderate-to-severe UC [9].

The first next-generation small molecule drug to be approved by the US Food and Drug Administration (FDA) and the European Medicine Agency (EMA) for the treatment of patients with moderate-to-severe ulcerative colitis was tofacitinib, a Janus kinase (JAK) inhibitor [10]. Phase 3 randomized clinical trials have shown that additional drugs, such as filgotinib and upadacitinib, are effective for the treatment of UC [11,12].

However, a large group of patients still has a history of inadequate response (primary failure) or loss of response (secondary failure) to therapies leading to surgery. For this reason, it is important to act on new immune pathways with new therapies [13,14].

**Figure 1:**
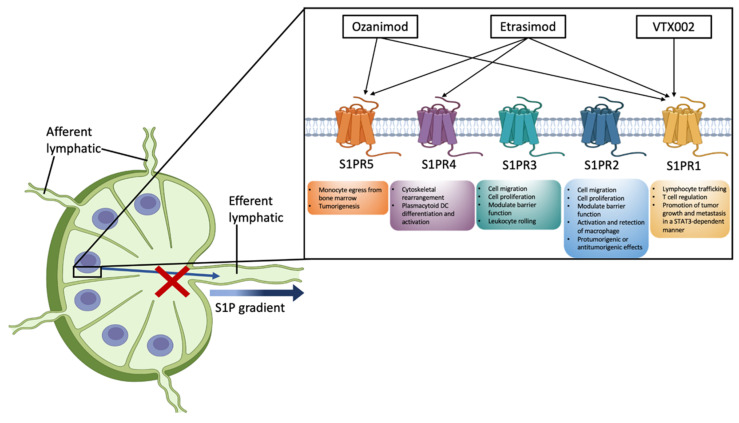
Selective action of ozanimod, etrasimod and VTX002 towards sphingosine 1-phosphate (S1P) receptors (S1PR1-5). S1P is a membrane-derived lysophospholipid widely expressed in all tissues. S1P plays a key role in regulating the trafficking of multiple immune cells, including T and B cells. The concentration of S1P is relatively low in lymphoid organs compared with the lymph, forming an S1P gradient that, in normal conditions, allows T and B cells to egress from the lymph node to peripheral tissues, activating immune response [33,34]. The biological effects of S1P are mediated via five specific G-protein-coupled receptors, S1PR1–5. S1PR1, S1PR2 and S1PR3 are ubiquitously expressed, and S1PR4 and S1PR5 have a more restricted expression in the hematopoietic and central nervous system, respectively [24]. S1PR1 activation is involved in lymphocyte trafficking, T cell regulation, and promotion of tumor growth and metastasis in a STAT3-dependent manner. S1PR2 and S1PR3 promote cell migration and proliferation and modulate barrier function. S1PR2 regulates macrophage activation and retention and exerts protumorigenic or antitumorigenic effects. S1PR3 also promotes leukocyte rolling. S1PR4 mediates cytoskeletal rearrangement and plasmacytoid dendritic cell differentiation and activation. S1PR5 is involved in monocyte egress from bone marrow and tumorigenesis [24,25,26]. S1PRs regulate immune cell trafficking, activation and differentiation, and for this reason, it has become a new target for the treatment of immune-mediated disease.

UC is characterized by the trafficking and gathering of autoreactive B and T lymphocytes. Sphingosine-1-phosphate (S1P) is a membrane-derived lysophospholipid signaling molecule involved in the modulation of the immune response (Figure 1). The inhibition of SP1, through the modulation of its receptors (S1P receptor subtypes 1–5; SP1R1–SP1R5) has demonstrated encouraging results in the treatment of Crohn’s disease (CD) and UC, and this evidence has proven that the S1P pathway could be considered a novel therapeutic target [15,16,17].

In fact, the S1PR modulator dampens the inflammatory response in UC by sequestering lymphocytes in the lymph nodes [18]. S1P functions as an extracellular signaling molecule by preferentially binding to five G-protein-coupled receptor subtypes (S1PR1–5) that are highly expressed in T cells and other cell types [19]. Most immune cells produce S1P, which is essential for lymphocyte homing to lymphoid organs and migration of lymphocytes from secondary lymphoid organs, thymus, and bone marrow into the blood [20]. B and T lymphocytes, dendritic cells, and endothelial cells all express the SP1R, which is important in the regulation of chronic inflammation and lymphocyte egress from peripheral lymphoid organs [21]. SP1R modulators sequester lymphocytes in the lymph nodes, resulting in fewer immune cells in the circulating blood to exacerbate inflammation [22,23].

The biological effects of S1P are mediated via five specific G-protein-coupled receptors, S1PR1–5. S1PR1, S1PR2, and S1PR3 are ubiquitously expressed, and S1PR4 and S1PR5 have a more restricted expression in the hematopoietic and central nervous systems, respectively [24]. S1PR1 activation is involved in lymphocyte trafficking, T cell regulation, and promotion of tumor growth and metastasis in a STAT3-dependent manner. S1PR2 and S1PR3 promote cell migration and proliferation and modulate barrier function. S1PR2 regulates macrophage activation and retention and exerts protumorigenic or antitumorigenic effects. S1PR3 also promotes leukocyte rolling. S1PR4 mediates cytoskeletal rearrangement and plasmacytoid dendritic cell differentiation and activation. S1PR5 is involved in monocyte egress from bone marrow and tumorigenesis [24,25,26].

Ozanimod, an S1PR modulator, became the second small molecule drug to receive approval for UC [27], even though guidelines for the treatment of moderate to severe UC do not yet include ozanimod [28,29].

Other S1PR modulator compounds, including etrasimod and VTX002, have been tested in phase 2 and 3 randomized clinical trials (RCTs) for the management of moderate to severe UC patients [30,31,32].

The aim of this review is to provide an overview of the efficacy and safety of S1PR modulators in the management of patients with moderate-to-severe UC.

## 2. Ozanimod

### 2.1. Mechanism of Action

Ozanimod, an oral selective S1P1R and S1P5R modulator is now approved by the FDA and EMA for the treatment of patients with moderate-to-severe UC [27]. It has a selectivity of 27 fold for S1PR1 in comparison with S1PR5 and of 10,000 fold over S1PR2-4 [16].

When the interaction of S1P with S1PR1 is prevented, activated T cells are retained in the lymph node and do not migrate into the tissues. So, the number of circulating lymphocytes is reduced, which in turn results in a reduction in tissue inflammation [35]. With low or no effect on effector memory cells, it has the greatest effects on lymphocytes that express the chemokine receptor 7, which include naive and central memory lymph node-homing cells, preserving some circulating protective immunity [36,37].

Ozanimod guarantees a durable but also rapidly reversible reduction in peripherical lymphocytes via internalization and degradation of S1P1R [16].

In experimental animal models of colitis caused by CD4+ CD45RBHi T-cell transfer and trinitrobenzene sulfonic acid, ozanimod decreased inflammatory markers (IFN-gamma, IL-10, IL-1B, IL-6, IL-8, TNF-alfa) [16].

### 2.2. Pharmacokinetics

Ozanimod is orally administered with a slow increase in plasma concentration with a Tmax of roughly 10 h [38]. Ozanimod showed linear pharmacokinetics with dose-proportional exposure and subject variability. Moreover, ozanimod steady-state with a high volume of distribution is achieved by day 7; this shows that the molecule is widely distributed into tissues with a 20-h elimination half-life, so a once-daily dosage is possible [39].

Renal clearance plays no significant role in the removal of ozanimod. Ozanimod undergoes two parallel metabolic pathways, which result in the formation of the major metabolite RP101988 and the minor metabolite RP101075 (both active metabolites with comparable in vitro S1P selectivity). Ozanimod is converted to RP101988 by alcohol- and aldehyde-dehydrogenase and RP101075 by CYP3A. Moreover, the two metabolites have linear pharmacokinetics (PK) with dose-proportional exposure and an elimination half-life of 19 to 22 h [40,41].

Ozanimod and its metabolites are not affected by food, including high- or low-fat meals, and can therefore be administered in either a fed or fasting condition [40].

P-glycoprotein (P-gp) is a weak substrate of ozanimod, whereas P-gp and the breast cancer resistance protein (BCRP) drug transporter are both substrates of RP101988. Drug–drug interaction investigations showed that CYP3A and P-gp inhibition (itraconazole) had no effect on ozanimod exposure, whereas cyclosporine, a potent P-gp and BCRP transporter inhibitor, raised total agonist exposure (ozanimod plus RP101988 and RP101075) by 50% and this could increase the risk of QTc prolongation [40,41].

Moreover, smoke has been investigated for having a role in drug concentration: smoking was not associated with a substantial difference in absolute lymphocyte count, so there is no loss of efficacy in smokers [42].

### 2.3. Efficacy and Safety

The efficacy of ozanimod in moderate-to-severe UC has been evaluated in the randomized, placebo-controlled, double-blind phase 2 trial TOUCHSTONE [43] (Table 1). In this study, a total of 197 patients were randomly assigned to three different groups in a 1:1:1 ratio. The first group received a daily dose of ozanimod at 0.5 mg (*n* = 65), the second group received a daily dose of 1 mg (*n* = 67), and the third group received a placebo (*n* = 65) for a duration of up to 32 weeks. The disease activity was measured using the Mayo Clinic score, which ranges from 0 to 12. The subscores range from 0 to 3, with higher scores indicating more severe disease. The primary objective of the study was to assess clinical remission at 8 weeks, defined as a Mayo Clinic score of ≤2 with no subscore exceeding 1. The secondary outcomes were valued at 8 weeks considering: clinical response (decrease in Mayo Clinic score of ≥3 points and ≥30% and decrease in rectal bleeding subscore of ≥1 point or a subscore ≤ 1), change from baseline in the Mayo Clinic score, and mucosal healing (endoscopy subscore ≤ 1). Exploratory outcomes included clinical response, clinical remission, mucosal healing, and change in the Mayo Clinic score at week 32 and histologic remission (Geboes score < 2, on a scale from 0 to 5, with higher scores indicating more severe histologic inflammation) at weeks 8 and 32.

Eligible patients were 18 to 75 years old and had UC, with a Mayo Clinic score of 6 to 12 and an endoscopic subscore of 2 or 3. Moreover, in this study, patients included were both biological-naive and on biological therapy, but patients receiving biologic agents were required to discontinue these agents 5 half-lives before starting the trial. 

In comparison to patients who received a placebo, 16% of patients who received 1 mg of ozanimod and 14% of those who received 0.5 mg of ozanimod achieved clinical remission at 8 weeks (*p* = 0.048 and *p* = 0.14, respectively, for the comparison of the two dosages of ozanimod with placebo). Because there were no significant differences in the primary outcome between the 0.5 mg of the ozanimod group and the placebo group, the hierarchical testing plan deemed the analyses of secondary outcomes exploratory. At 8 weeks, 57% of those getting 1 mg of ozanimod and 54% of those receiving 0.5 mg experienced a clinical response as opposed to 37% of those receiving the placebo (*p* = 0.02 and *p* = 0.06, respectively). At week 32, the rates of clinical remission were 21% in the ozanimod treatment group receiving 1 mg, 26% in the ozanimod treatment group receiving 0.5 mg, and 6% in the placebo treatment group (*p* = 0.01 and *p* = 0.002, respectively); the rates of clinical response were 51% (*p* < 0.001), 35% (*p* = 0.06), and 20%, respectively. 

Efficacy data were confirmed in the Open-label Extension (OLE) of the TOUCHSTONE study in which 170 patients were followed for up to 200 weeks [44]. Patients with moderately to severe UC (mayo score 6–12 with an endoscopic subscore of ≥2) who enrolled in the main study could enter the optional OLE and receive treatment with ozanimod HCl 1mg orally once daily. Of these, 81 patients entered the OLE period at the end of the induction period, seven entered during the maintenance period, and 82 entered at the end of the maintenance period. At the time of the analysis, 99 patients [58%] had discontinued the OLE study, with 28% of the patients discontinuing in the first year and an annual discontinuation rate of 15–18% for existing patients in years 2–4. During the OLE period, the study’s goals were to evaluate the long-term safety and effectiveness of ozanimod HCl 1 mg daily. Observed cases [OC] and non-responder imputation [NRI] were used to summarize descriptively the partial Mayo score [pMS] clinical response [(reduction from baseline in pMS of ≥2 points and ≥30% and either a reduction in rectal bleeding score (RBS) of ≥1 point or an absolute RBS of ≤1 point)] and remission [pMS of ≤2 points with no individual subscore of >1 point] through OLE week 200. At OLE week 56 and at the end of the course of therapy, an endoscopy was necessary. The endoscopy-related parameters were analyzed and reported at weeks 56 and 104 using the observed cases (OC) analysis. Additionally, the parameters were summarized at week 56, utilizing the non-responder imputation (NRI) analysis. At OLE week 200, the partial Mayo measures showed clinical response rates of 93.3% and remission rates of 82.7% based on the OC analysis. The more conservative NRI analysis yielded lower rates of 41% for clinical response and 37% for remission. At weeks 56 and 104, respectively, histological remission (Geboes index score of <2.0) rates were 46.3% and 38.5%, and endoscopic improvement (endoscopic subscore of ≤1 point) rates were 46.4% and 46.5% [OC]. 

Consistent results were obtained in phase 3, a multicenter, randomized, double-blind, placebo-controlled trial of ozanimod as induction and maintenance therapy in patients with moderately to severely active UC (True North) (Table 1) [45]. 

This trial enrolled 1012 patients. During the 10-week induction period, patients were divided into two cohorts. Cohort 1 (*n* = 645) received either 1 mg of oral ozanimod hydrochloride (*n* = 429) or a placebo (*n* = 216) once daily in a double-blind manner. Meanwhile, cohort 2 (*n* = 367) received open-label ozanimod at the same daily dose. After 10 weeks, patients from either cohort who demonstrated a clinical response to ozanimod underwent randomization again. They were assigned to receive either double-blind ozanimod or placebo for the maintenance period, which extended up to week 52. The primary endpoint assessed during both the induction and maintenance periods was the percentage of patients achieving clinical remission. This assessment was based on the three-component Mayo score. Clinical remission was defined as a rectal-bleeding subscore of 0; a stool-frequency subscore of 1 or less, with a decrease of at least 1 point from baseline; and an endoscopy subscore of 1 or less. The secondary endpoints for the induction period (at week 10) were the percentages of patients with clinical response (based on the three-component Mayo score), endoscopic improvement (defined as a mucosal endoscopy subscore of ≤1 without friability), and mucosal healing (endoscopic improvement plus histologic remission, defined as a mucosal endoscopy score of ≤1 and a Geboes score of <2.0). The secondary endpoints for the maintenance period (at week 52) were the percentages of patients with a clinical response, endoscopic improvement, maintenance of clinical remission (remission at week 52 in the subgroup of patients with remission at week 10), glucocorticoid-free remission (remission with no glucocorticoid use for ≥12 weeks), mucosal healing, and durable clinical remission (remission at weeks 10 and 52, assessed in all patients in the maintenance period).

Patients had to be between the ages of 18 and 75 and have moderately to severely active UC, which was indicated by a Mayo score between 6 and 12, an endoscopic subscore between 2 and 6, a rectal bleeding subscore between 1 and 2, and a stool frequency subscore between 1 and 2. Patients having a clinically significant cardiac condition, a history of uveitis or macular edema, or who had not responded to induction therapy with at least two biologic drugs licensed for the treatment of UC were excluded from the trial.

During the induction period, a total of 645 individuals were enrolled in Cohort 1, and 367 individuals were enrolled in Cohort 2. For the maintenance period, 457 patients were included. Both during induction (18.4% vs. 6.0%, *p* < 0.001) and maintenance (37.0% vs. 18.5% (among patients with a response at week 10), *p* < 0.001), individuals receiving ozanimod more frequently experienced clinical remission than those receiving placebo. Ozanimod significantly increased the likelihood of clinical response for both induction (47.8% vs. 25.9%, *p* < 0.001) and maintenance (60.0% vs. 41.0%, *p* < 0.001) compared to placebo. Ozanimod significantly outperformed the placebo in both periods for all other secondary outcomes.

Regarding safety, considering the TOUCHSTONE study, no important differences were observed among patients who received ozanimod at the dose of 0.5 or 1 mg or placebo in the most commonly reported adverse events during the trial [Total adverse events (TAEs) 40% vs. 39% vs. 40%, respectively)] (Table 1) [43]. During the induction period, one patient in the 0.5-mg ozanimod group had a first-degree atrioventricular block and sinus bradycardia (1.5% vs. 0 in placebo) with evidence of preexisting bradycardia. After these occurrences, the patient stopped receiving treatment. There was no evidence of episodes of atrioventricular block during the maintenance period. Moreover, there were no described episodes of macular edema either in induction or in the maintenance period. Absolute lymphocyte counts in the blood fell by a mean of 32% in patients who got 0.5 mg of ozanimod and by 49% in those who received 1 mg of ozanimod from baseline to week eight. At week 8, a total of 30% of patients in the ozanimod 0.5 mg group and 53% of patients in the ozanimod 1 mg group had absolute lymphocyte counts that were below the lower limit of the normal range. During treatment, the alanine aminotransferase level in four patients who got ozanimod [one patient received 0.5 mg, and three patients received 1 mg (1.5% vs. 4.5%)] increased by more than three times the upper limit of the normal range.

In the OLE of TOUCHSTONE, where ozanimod was given at a dose of 1 mg for 200 weeks, adverse events were monitored throughout the study [44]. The most frequent treatment-emergent adverse events (TEAEs) were upper respiratory infections (5.9%), hypertension (5.9%), UC (6.5%), and elevated gamma-glutamyltransferase (5.3%). Due to retinal vein thrombosis, two participants had macular edema or thickness; however the condition did not cause the trial to be stopped. The elevation of alanine aminotransferase was registered in 3.5% of patients. A TEAE of lymphopenia or decreased lymphocyte counts occurred in nine patients (5.3%); these events were deemed mild in two cases, moderate in six cases, and severe in one case. The mean absolute blood lymphocyte counts generally declined from OLE baseline (1.910 × 10^9^ cells/L, SD = 0.855) to OLE week 4 (0.990 × 10^9^ cells/L; SD = 0.552) and then remained stable after that. However, only one case of lymphopenia was classified as severe but was not associated with infection and did not result in study discontinuation. During the OLE phase, three patients experienced a serious infection. However, none of these infections were associated with grade 4 lymphopenia, which is characterized by an absolute lymphocyte count of less than 200. The most commonly reported serious adverse events (SAEs) were UC (3.5%), anemia (1.5%) and ischemic stroke (1.5%). There were no significant irregularities in the cardiac chronotropic or negative effects on the conduction of the heart. TEAEs brought to study drug discontinuation during the OLE were indicated for 17 patients (10%).

Safety was also assessed in phase 3, a multicenter, randomized, double-blind, placebo-controlled trial of ozanimod as induction and maintenance therapy in patients with moderately to severely active UC (True North) (Table 1) [45]. During the maintenance period, the number of adverse events was higher in the ozanimod group than in the placebo group (49.1% vs. 36.6%), but it was comparable in both groups during the induction period (38% in the placebo group in cohort 1, 40.1% in the ozanimod group in cohort 1 and 39.8% in the ozanimod group in cohort 2). During the induction period, the overall incidence of non-serious infection with ozanimod treatment was comparable to that with placebo (11.6% in placebo group in cohort 1, 10.7% in ozanimod group in cohort 1 and 12.5% in ozanimod group in cohort 2), but it was greater during the maintenance period (23.0% vs. 11.9%). Less than 2% of severe infections occurred in each group. In individuals who got ozanimod, the absolute lymphocyte count fell by a mean of about 54% from baseline to week 10. During the induction period, 1.1% of the patients who received ozanimod (in cohorts 1 or 2) and none of the patients who received placebo experienced absolute lymphocyte counts of fewer than 200 cells per cubic millimeter. Throughout the entire 52-week trial, a total of 17 patients initially had an absolute lymphocyte count below 200 cells per cubic millimeter. However, following the initiation of ozanimod treatment, their lymphocyte counts subsequently increased and consistently maintained a level at or above 200 cells per cubic millimeter. Absolute lymphocyte counts of greater than 200 cells per cubic millimeter were present in none of the patients with severe or opportunistic infections. Bradycardia was more frequent during the induction phase of ozanimod therapy compared to placebo (0.5% in cohort 1, 0.8% in cohort 2 vs. 0% in placebo), but not during the maintenance phase. There were no instances of third-degree or second-degree type 2 atrioventricular blocks. Ozanimod treatment was more frequently associated with elevated liver aminotransferase levels than placebo. In 3 of 796 patients (0.4%) during the induction period and in 1 of 230 patients (0.4%) during the maintenance period, abnormal liver function tests resulted in the termination of ozanimod therapy. Three patients using ozanimod experienced macular edema (*n* = 2 in the induction period: n = 1 in cohort 1 (0.2%), *n* = 1 in cohort 2 (0.3%) and *n* = 1 in the maintenance period (0.4%)); all cases improved when the medication was stopped.

## 3. Etrasimod

### 3.1. Mechanism of Action

Etrasimod is an oral, full agonist of S1P1, S1P4, and S1P5 receptors in development for the treatment of immune- and inflammatory-mediated diseases. Additionally, it was demonstrated that etrasimod has an inhibitor effect on pro-inflammatory cytokines, such as TNF-α, IL-1β, IL-6, and IL-17A and increases the effect of anti-inflammatory cytokine IL-10 [46].

### 3.2. Pharmacokinetics

Etrasimod is orally administrated, and it is absorbed and metabolized with no clinically significant pharmacokinetic variation based on sex, age, body weight, or race [47].

The median time to achieve the highest plasma concentrations following fast absorption is 6 to 8 h. With a consistent mean elimination half-life of 26.2–32.5 h following a single dose of etrasimod, its pharmacokinetics are dose-proportional and reach steady-state by day 7 [48]. A healthy distribution in tissue is indicated by the apparent volume of distribution being two times that of the total body water [49]. Etrasimod has a sluggish rate of elimination and undergoes significant cytochrome P450 oxidation, dehydrogenation, sulfation, and glucuronidation.

Etrasimod and its related metabolites are primarily eliminated through hepato-biliary excretion; renal clearance of etrasimod is negligible. Etrasimod’s various biotransformation pathways and multiple CYPs (mostly CYP2C8 and CYP2C9) are involved in reducing the likelihood of PK drug–drug interactions [50].

### 3.3. Efficacy and Safety

The phase II OASIS trial was a double-blind, parallel-group, placebo-controlled that randomly assigned 156 patients with ulcerative colitis to treatment with once-daily etrasimod 1 mg (*n* = 52) or 2 mg (*n* = 50) or placebo (*n* = 54) (Table 2). The primary endpoint was the improvement in the three-component (endoscopic score, rectal bleeding, and stool frequency) modified Mayo Clinic score (mMCS) at week 12, analyzed by using the multiple imputation method for missing data. The difference in mean change from baseline between patients treated with 2mg etrasimod versus placebo was −0.99 points (90% CI 0.30–1.68; *p* = 0.009), while in patients treated with 1mg etrasimod, there was no significant difference versus placebo (−0.43 points, 90% CI −0.24 to +1.11; *p* = 0.146). Endoscopic improvement, defined as a Mayo Clinic endoscopic subscore of ≤1 point, was also met by a greater proportion of patients when etrasimod 2 mg was prescribed (41.8% vs. 17.8% for placebo; *p* = 0.003). Even in this endpoint, there was no significant difference between etrasimod 1 gr vs. placebo (22.5% vs. 17.8%; *p* = 0.306). Results of exploratory efficacy endpoints at week 12 showed clinical remission (Mayo Clinic endoscopic subscore ≤ 1 [with the absence of friability], rectal bleeding score ≤ 1, and stool frequency score ≤ 1 with a frequency decrease of ≤1 point from baseline) in 50 (33%) patients in the etrasimod 2 mg group vs. 54 (8.1%) in the placebo group (*p* < 0.001) and clinical response (met the criteria for clinical remission or had a decrease in modified MCS of ≤2 points and a decrease in rectal bleeding of ≤1) in 50 (50.6%) vs. 54 (32.5%), in etrasimod 2 gr and placebo group, respectively (*p* = 0.028) [30].

Efficacy data were confirmed in the Open-label Extension (OLE) of the OASIS study 112 patients were enrolled to receive etrasimod 2 mg, while six patients received a placebo. Once daily etrasimod 2 mg was administered, irrespective of their treatment assignment or response in the double-blind study, for up to an additional 34 to 40 weeks. The overall group consisted of patients who received any treatment, including placebo, etrasimod 1 mg, or etrasimod 2 mg, during the double-blind study. The etrasimod 2 mg treat-through group received etrasimod 2 mg during both the double-blind study and OLE. Results showed that 64% [72/112] of patients had a clinical response, 33% [37/112] were in clinical remission, and 43% [48/112] had endoscopic improvement. Additionally, patients who were previously treated with biologics showed worst responses than naive ones (clinical response 73.9% vs. 80.3%; clinical remission 30.4% vs. 42.6%; endoscopic improvement 54.2% vs. 49.2%) [51].

Important results were also confirmed in two randomized, multicenter, double-blind, placebo-controlled, phase 3 trials ELEVATE UC 52 and ELEVATE UC 12 (Table 2) [31]. Clinical remission was the primary endpoint in both studies, intended as stool frequency subscore = 0 or stool frequency subscore = 1 with a ≥1-point decrease from baseline, rectal bleeding subscore = 0, and endoscopic subscore of 1 or less, without friability. Some of the secondary endpoints were endoscopic improvement (endoscopic subscore ≤ 1, without friability), symptomatic remission (stool frequency subscore = 0 or stool frequency = 1 with a ≥1-point decrease from baseline and rectal bleeding subscore = 0), and histological remission (endoscopic subscore ≤ 1, without friability, with histological remission, intended as Geboes Index score < 2.0).

In ELEVATE UC 52, at the 12-week induction period, 74 (27%) of 274 patients in the etrasimod group achieved the primary endpoint, compared with patients in the placebo group (10 (7%) of 135 patients; *p* < 0.0001). At the end of the maintenance period, at week 52, 88 (32%) of 274 patients vs. 9 (7%) of 135 patients reached the primary endpoint (*p* < 0.0001). Patents naive from biologic had better response even in this case [60 (31%) of 194 vs. 14/80 (18%) at 12 weeks; 71 (37%) of 194 vs. 17 (21%) of 80 at 52-week, in etrasimod and placebo group, respectively]. Patients in the etrasimod group had a significant endoscopic improvement towards placebo (96 (35%) vs. 19 (14%; *p* < 0.0001) at week 12, 94 (33%) vs. 11 (8%; *p* < 0.0001) at week 52). Symptomatic and histological remission were also met [126 (46%) vs. 29 (21%; *p* < 0.0001) and 58 (21%) vs. 6 (4%; *p* < 0.0001) at week 12; 113 (39%) vs. 19 (13%; *p* < 0.0001) and 127 (44%) vs. 28 (19%; *p* < 0.0001) at week 52].

During the ELEVATE UC 12 study, 55 out of 222 patients (25%) in the etrasimod group achieved clinical remission after the 12-week induction period. In contrast, only 17 out of 112 patients (15%) in the placebo group achieved clinical remission within the same period. The observed difference in remission rates between the two groups was statistically significant, with a *p*-value of 0.026. All the secondary endpoints were reached vs. placebo [endoscopic improvement: 68 (31%) vs. 21 (19%; *p* = 0.0024); symptomatic remission: 104 (47%) vs. 33 (29%; *p* = 0.0047); histological remission: 36 (16%) vs. 10 (9%; *p* = 0.012)].

Regarding safety, in the OASIS II trial (Table 2), 56% of patients treated with etrasimod 2mg reported one or more TEAEs, the most common ones where UC worsening and electrocardiogram T-wave abnormal, leading to discontinuation of the study in 2 (4%) and 1 (2%), respectively. In total, ten serious treatment-emergent adverse events (TEAEs) occurred in nine patients, which account for 5.8% of all patients involved in the study. Out of these, three patients were receiving etrasimod 1 mg, and six patients were on the placebo. Additionally, nine TEAEs resulted in the discontinuation of the study drug in seven patients, comprising 4.5% of all patients. Among them, three patients were taking etrasimod 1 mg, and four patients were taking etrasimod 2 mg. The majority of the reported TEAEs (75%) were categorized as mild to moderate in severity [30].

In the OLE trial, the most reported TEAEs in patients treated with etrasimod 2 mg in the OLE were worsening UC (21/112 [19%] patients) and anemia (12/112 [11%] patients). Fourteen serious TEAEs were reported in seven patients treated with etrasimod 2 mg in the OLE. Ten of 112 [9%] patients in the etrasimod 2 mg safety population discontinued the study drug due to a TEAE [eight patients with worsening UC and one patient each with atrial fibrillation and headache] [51].

In the ELEVATE UC 52 trial, AEs were reported in 206 out of 289 patients (71%) in the etrasimod group, compared to 81 out of 144 patients (56%) in the placebo group. On the other hand, in the ELEVATE UC 12 trial, 112 out of 238 patients (47%) in the etrasimod group experienced AEs, while 54 out of 116 patients (47%) were in the placebo group (Table 2). There were no statistical differences between the etrasimod and placebo group in terms of AEs leading to treatment discontinuation (12 (4%) of 289 patients vs. (5%) of 144 patients in ELEVATE UC 52; 13 (5%) of 238 patients vs. (1%) of 116 patients in ELEVATE 12). The incidence of serious adverse events was found to be low and comparable between the etrasimod and placebo groups in both studies. In ELEVATE UC 52, there were 20 cases (7%) of serious adverse events among the 289 patients in the etrasimod group and nine cases (6%) among the 144 patients in the placebo group; six [3%] of 238 patients in the etrasimod group vs. two [2%] of 116 patients in the placebo group in ELEVATE UC 12). Atrioventricular block, first and second degree, and macular edema represented <1% of AEs in all RCTs about etrasimod. No deaths or malignancies were reported [31].

## 4. VTX 002

VTX 002 (OPL 002) is an oral, highly selective sphingosine 1 phosphate receptor 1 (S1P1R) modulator in development for UC. The highest development phase is phase II: the 26th of January, 2023 was published interim pharmacodynamics data from a phase II trial in UC.

### 4.1. Mechanism of Action

VTX002, by blocking only the S1P1 receptor function, leads to internalization of S1P1R, ubiquitination and proteasome degradation of the receptor, producing sustained lymphopenia and subsequently reduction of lymphocytes access to inflammation’s sites [52]. His selectivity for S1P1R allows him to not interfere with other S1P receptors located in the central nervous system or in the heart [53].

VTX 002 determines a potent and sustained lymphocyte reduction and a rapid on-and-off rate. In fact, in Phase 1, multiple ascending dose (MAD) studies, once-daily dosing of OPL-002 led to a dose-dependent steady-state reduction in absolute lymphocyte count of up to 65%. After the last dose of OPL-002, lymphocyte counts returned to normal by 72 h [32].

### 4.2. Pharmacokinetics

In a Phase 1 study, VTX002 was administered orally as a single dose in 6 cohorts (SAD) or daily administration for 21–28 days (inclusive of 7 days of dose titration) in 5 cohorts (MAD), with each cohort randomized to OPL-002 (*n* = 6) or placebo (*n* = 2). The study shows that single and multiple doses of VTX002 were associated with dose-dependent and linear PK with no food effect observed. The PK profile was characterized by a Tmax of 1.8 to 3.67 h. Terminal half-life after both single and multiple doses ranged from 16 to 23 h [32].

VTX002 was tested as a spray-dried dispersion (SDD) oral suspension in a phase 1 study in healthy participants and was well-tolerated up to 45 mg. Another phase 1 randomized study on 12 healthy adults compared pharmacokinetics (PK) and pharmacodynamics of the SDD oral suspension and a tablet formulation of VTX002: one group was receiving single oral doses of 5 mgVTX002 tablets, the other group 20 mg VTX002 tablets and the last group 20 mg VTX002 SDD suspension followed by at least a 1-week washout between each dose. In the end, all doses and formulations of VTX002 were well-tolerated and associated with absolute lymphocyte count reductions. So, these data enable tablet dose selection for a phase 2 study of VTX002 for treatment of UC that is underway [54].

Furthermore, studies proved that VTX002 has very low central nervous system penetration and ocular distribution (low risk of macular edema), no CYP450 interactions (fewer potential drug–drug interactions) and no active metabolites (low risk of low liver injury) [32].

### 4.3. Efficacy and Safety

There are limited data about the efficacy and safety of VTX 002. In a randomized, double-blind, placebo-controlled phase 1 trial involving 88 subjects, VTX002 was administered orally as a single dose in 6 cohorts (SAD) or daily administration for 21–28 days (inclusive of 7 days of dose titration) in 5 cohorts (MAD), with each cohort randomized to OPL-002 (*n* = 6) or placebo (*n* = 2). During the study, the drug exhibited excellent tolerance when administered once daily to healthy subjects for a period of up to 28 days. There were no reports of serious adverse events, and none of the subjects showed any signs of liver function test elevations, pulmonary function or ocular exam abnormalities, or any other noteworthy safety concerns. Additionally, there was no clinically significant reduction in heart rate after the first dose during the 28-day treatment period. In the MAD study, where doses were escalated up to 45 mg after a 7-day dose titration phase, the maximum decrease in heart rate from the baseline, following the initial dose at the target level, was less than four beats per minute [32].

Currently, there is an ongoing multicenter, randomized, double-blind, placebo-controlled, phase 2 study designed to evaluate both the clinical efficacy and safety of VTX002 in individuals with moderately to severely active UC. The estimated primary completion date is around October 2023. The study will enroll 180 patients, who, after 28 days of the screening period, will start a 13-week double-blind period: participants will receive either active Dose A, Dose B or Placebo; secondarily, there will be a Long-Term Extension (LTE) treatment period that will last up to 39 weeks; this will be followed by an Open-Label Extension (OLE) treatment period, which will extend up to 143 weeks [55].

## 5. Discussion

Immune cell trafficking is a crucial component of the intestinal immune response [56,57,58,59,60,61,62]. Adhesion molecules, chemoattractants, receptors on immune cell surfaces, blood vessels, and stromal intestinal tissue, as well as signaling pathways, particularly those S1P-modulated ones, are all involved in this process. Inhibiting lymphocyte egress from the lymph nodes to the bloodstream may be a safe and effective alternative mechanism to reduce inflammation in UC, according to promising preclinical and clinical data with different subtypes of oral S1PR modulators [63].

S1P plays a fundamental role in the immune system. It has been evaluated as a therapeutic target for several immune-mediated disorders beyond UC, such as multiple sclerosis (MS) [64,65], psoriasis [66], rheumatoid arthritis [67], systemic lupus erythematosus [68]. In fact, ozanimod has been authorized for clinically isolated syndrome, relapsing-remitting MS by the EMA and for active secondary progressive MS by the FDA [27]. Other S1PR modulator approved for MS are fingolimod [69], siponimod [70] and ponesimod [71]. Ponesimod was also evaluated, in a phase 2 study, as therapy in patients with moderate to severe chronic plaque psoriasis [72]. This study demonstrated a 75% reduction in affected areas and disease severity index in 77% of patients. Moreover, amiselimod is an S1PR modulator that has been evaluated for the therapy of systemic lupus erythematosus [68] with promising results. In addition, cenerimod is another S1PR modulator assessed in patients with active systemic lupus erythematosus, but data are not yet available [73].

The only S1PR modulator approved for UC is ozanimod [27]. A phase III trial, True North, has shown the efficacy of this small molecule towards placebo in both induction and maintenance therapy. Almost 18% of patients reached clinical remission at induction and 37% at maintenance. Endoscopic remission was achieved in 27% and 46% of the induction and maintenance period, respectively [45]. These results were not different from the literature about other available molecules for the treatment of moderate–severe UC [6,7,8]. The True North demonstrated that biologic naive patients had significantly greater response and remission rates than patients who previously received other advanced therapy; there are other ongoing real-life studies in support of this finding [45].

The advantage of ozanimod is the oral administration and its rapid mechanism of action by reducing the absolute lymphocyte count in the blood. On the other hand, interrupting the drug brings a rapid normalized count of peripheral lymphocytes [42].

Nevertheless, ozanimod has been associated with AEs, such as bradycardia, macular edema and elevation of liver enzymes [43]. To date, there is no validated screening or follow-up to prevent these AEs. An electrocardiogram should be recommended in patients with suspected or previous history of bradycardia or other heart disorders. In comparison, a multidisciplinary discussion with ophthalmologists should be conducted in patients with ocular comorbidity. More studies are needed to discover the real criteria to identify the patient who can be fit for this drug.

Data on the long-term efficacy and safety of ozanimod from the ongoing True North open-label extension are now available [74]. A total of 131 patients started the OLE after 52 weeks of continuous ozanimod treatment showing clinical improvement; at the time of the data cutoff, 87.0% had finished OLE Week 46, and 71.8% had finished OLE Week 94. The majority (68%) had left-sided UC disease, while 32% had previously used tumor necrosis factor inhibitors, and 24% concurrently used corticosteroids at baseline True North. According to this interim analysis of the True North OLE, the majority of patients who had a clinical response after taking ozanimod for a year continued to benefit for an additional two years. Moreover, with prolonged ozanimod use, no new safety data were found.

Of note, a reliable safety profile of ozanimod was also shown in MS. SUNBEAM [65] is an RCT which was assessed the safety and efficacy of ozanimod versus intramuscular interferon beta-1a in subjects treated for at least 12 months. In this study, there were no first-dose, clinically significant bradycardia or second-degree or third-degree atrioventricular block; the incidence of serious adverse events was low and similar across treatment groups. There were no serious opportunistic infections occurred in ozanimod-treated participants. Moreover, in RADIANCE [64], a phase III trial that enrolled 1320 active relapse-remitting MS patients, most of the adverse events were mild or moderate: in more than 5% of patients treated with ozanimod occurred nasopharyngitis, pharyngitis, urinary tract infections, and increased ALT and γ-glutamyl transferase levels; during dose escalation. There were no clinically meaningful cardiac findings and no serious opportunistic infections. These demonstrations can also help prove the safety of ozanimod in patients with UC.

While ozanimod proved to be effective in UC, it is not yet included in ECCO guidelines. The current standard of care for UC involves a stepwise approach, with the selection of medications based on the severity and extent of the disease [28]. There is little comparative data between ozanimod and other available drugs. Recently, the efficacy and safety of ozanimod, adalimumab, and vedolizumab in UC were indirectly compared [75]. Ozanimod significantly outperformed adalimumab in terms of clinical response (odds ratio [OR]: 1.53; *p* < 0.05) and endoscopic improvement (OR, 1.66; *p* < 0.05). No differences between ozanimod and vedolizumab were found in terms of clinical remission, clinical response, and endoscopic improvement. Additionally, antisphingosine therapy was associated with considerably decreased rates of infectious adverse events compared to anti-TNF during the induction phase (risk difference, −8.9%; *p* < 0.01) and to anti-integrin during the maintenance phase (risk difference, −46.3%; *p* < 0.01). The efficacy and safety of ozanimod is consistent with other drugs. More information is required to determine how to position ozanimod in the UC treatment algorithm, as well as the drug’s long-term effectiveness and safety in a real word setting.

Based on the available evidence, it is reasonable to hypothesize that ozanimod may be used both as first-line therapy or after the failure of other molecules.

Nowadays, data about the use of S1PR modulators in specific populations are limited. ECCO guidelines recommend that S1PR modulators should be avoided during pregnancy [76]. Similarly, there are still no data on their efficacy in patients with extraintestinal manifestations.

SP1R modulators have several advantages compared to biological drugs. Their oral administration could be focal for the patient’s therapy compliance: the STEPSTONE trial proved that oral administration once daily of this class of medications benefits drug compliance at least in the first 12 weeks of treatment [77]. In addition, unlike biologics, SP1R modulators are not immunogenic. Immunogenicity is associated with drug discontinuation, and the use of immunosuppressant drugs is often indicated to reduce the risk of producing autoantibodies, increasing the risk of adverse events [78].

Ozanimod has also been tested for the management of patients with CD. Promising results were obtained from the STEPSTONE trial, an open-label, phase II induction study about ozanimod in moderate-to-severely active CD [77]. A phase III multicenter, randomized, double-blind, placebo-controlled study of ozanimod as induction and maintenance therapy for moderate to severe active CD is ongoing and will provide further evidence about the efficacy and safety of this drug in CD [79,80,81].

Other S1PR modulators are currently undergoing clinical development for UC, but they are not yet approved by FDA or EMA. Etrasimod selectively targets S1PR 1 and 4, while VTX002 acts on S1PR 1.

The efficacy of etrasimod was proved in phase II and III trials, achieving higher rates of clinical remission, clinical response, endoscopic improvement and corticosteroid free-remission compared with placebo [30,31,51]. Both in preclinical and clinical studies, etrasimod was generally considered safe. In the OASIS trial phase II, three patients experienced asymptomatic conduction abnormalities, although pre-dose AV block was documented prior to treatment in all three patients. In all trials, the incidence of macular edema was low, as well as serious infections. No malignancies were reported [51]. Although no clinically relevant lymphopenia signal was observed in the etrasimod trials, in both ELEVATE studies, absolute lymphocyte counts returned to the normal range for most of the patients within 2 weeks after treatment discontinuation. Importantly, etrasimod could be used in a setting where the immune reconstitution is critical, differently from other S1PR modulators [31].

Also, there is an ongoing OLE study in which the long-term safety and effectiveness of etrasimod for UC will be assessed [82]. Patients who received either placebo or etrasimod were divided into two cohorts for analysis: those who received either placebo or etrasimod (the placebo-controlled cohort) and those who received ≥1 dose of etrasimod (all UC cohort). Preliminary data show that rates of AEs, serious AEs, and infections (including serious infections, opportunistic infections, and herpes zoster) are comparable across treatment groups and across the placebo-controlled and all UC cohorts. In the placebo-controlled group, bradycardia events were reported by 11 (1.8%) of the etrasimod-treated patients but not by the placebo-treated patients (9 of the 11 occurrences were asymptomatic). Other AEs of particular significance, such as high blood pressure and retinal edema, were comparable across treatment groups and all UC cohorts. Etrasimod was generally well tolerated and had an acceptable safety profile in individuals with moderately to highly active UC; these characteristics did not appear to change with the longer-term treatment of up to 2.5 years.

Conversely, more data are needed, especially about the pharmacokinetics, safety, and efficacy of VTX002. Currently under phase II RCT, this new drug has demonstrated a strong pharmacodynamic signal (high lymphocyte lowering and rapid lymphocyte recovery), combined with safety and tolerability, that could be a promising new potential treatment for IBD.

If we have very promising data on efficacy, the safety profile of these drugs still raises some doubts. Long-term data are lacking, and therefore, it is necessary to continue monitoring the treated patients to evaluate the real impact of these new molecules. However, a distinction must be made. In fact, ozanimod is already approved and used in clinical practice in many countries. Instead, etrasimod and VTX-002 are still being tested and will need further confirmation.

If etrasimod and VTX002 are approved, head-to-head trials between different S1PR modulators and between S1PR modulators and biological drugs/small molecules will be needed to compare effectiveness and safety and to provide further insights about the positioning of these drugs in the treatment algorithm of UC. Since the differences in the mechanism of action between the S1PR modulators (ozanimod S1PR 1,5, etrasimod S1PR 1,4,5, VTX002 S1PR1), they can have different specific impacts on immune cell trafficking, immune responses, and potential side effects that can determine its therapeutic potential and overall safety profile. However, it should be noted that it is not yet known whether drug selectivity will play a role in treatment decisions and whether the action on only one receptor (VTX) will have a benefit over drugs that act on two receptors (ozanimod) or more (etrasimod).

Considering the rapid pharmacokinetics and the safety profile of S1PR modulators, it could be reasonable to use them in dual therapy. The rationale for combining S1PR modulators with biologics, for example, is to potentially enhance the effectiveness of treatment by targeting multiple pathways involved in the inflammatory process. S1PR modulators can reduce immune cell trafficking [22], while biologics can directly inhibit specific inflammatory molecules [83]. The specific combination and sequencing of medications in dual therapy can vary depending on factors such as disease severity, treatment response, and individual patient characteristics. In addition, large prospective studies are needed to define the feasibility of this strategy and define the timing of dual therapy and the best combination of drugs.

## 6. Conclusions

S1P receptor modulators are effective and safe drugs for UC, as demonstrated by the available trials. Nowadays, ozanimod is the only drug in this category approved by FDA and EMA [27]. Ozanimod is an effective and well-tolerated treatment to both induce and maintain disease remission in UC. Further studies on S1PR modulators, including head-to-head trials and studies assessing their cost-effectiveness, are needed to position ozanimod in the treatment algorithm of patients with moderate-to-severe UC.

## Figures and Tables

**Table 1:** Summary of efficacy and safety of Ozanimod for Ulcerative Colitis.

Trial	Intervention		Primary Endpoint		Secondary Endpoints							Adverse Events		
**Touchstone** Phase 2 (*n* = 197) randomized, double-blind, placebo-controlled phase 2 trial			**Clinical remission**		**Clinical response**		**Mucosal healing**		**Histologic remission**			**Total adverse events** ***n* (%)**		**Serious adverse events** ***n* (%)**
			**Induction** (at 8 weeks) ***n* (%)**	**Mantainance** (at 32 weeks) ***n* (%)**	**Induction** (at 8 weeks) ***n* (%)**	**Mantainance** (at 32 weeks) ***n* (%)**	**Induction** (at 8 weeks) ***n* (%)**	**Mantainance** (at 32 weeks) ***n* (%)**	**Induction** (at 8 weeks) ***n* (%)**		**Mantainance** (at 32 weeks) ***n* (%)**			
	**Ozanimod 0.5 mg once daily** (*n* = 65)		9 (14) *p* = 0.14	17 (21) *p* = 0.002	35 (54) *p* = 0.06	23 (35) *p* = 0.06	18 (28) *p* = 0.03	21 (32) *p* = 0.006	9 (14) *p* = 0.63		15 (23) *p* = 0.02	26 (40)		1 (2)
	**Ozanimod 1 mg once daily** (*n*= 67)		11 (16) *p* = 0.048	14 (26) *p* = 0.01	38 (57) *p* = 0.02	34 (51) *p* < 0.001	23 (34) *p* = 0.002	22 (33) *p* = 0.005	15 (22) *p* = 0.07		21 (31) *p* < 0.001	26 (39)		3 (4)
	**Placebo** (*n* = 65)		4 (6)	6 (6)	24 (37)	13 (20)	8 (12)	8 (12)	7 (11)		5 (8)	26 (40)		6 (9)
**True North** Phase 3 (*n* = 1012) multicenter, randomized, double-blind, placebo-controlled phase 3 trial			**Clinical remission** ***n* (%)**		**Clinical response** ***n* (%)**		**Endoscopic improvement** ***n* (%)**		**Mucosa healing** ***n* (%)**	**Durable** **Remission** ***n* (%)**	**Glucocorticoid-free** **Remission** ***n* (%)**	**Maintenance of Remission** ***n* (%)**	**Total adverse events** ***n* (%)**	**Serious adverse events** ***n* (%)**
	**Induction **(10 weeks) **Cohort 1** ***n* = 645** (double-blind manner)	**Ozanimod 1 mg once daily** (*n* = 429)	79 (18.4) *p* < 0.001		205 (47.8) *p* < 0.001		117 (27.3) *p* < 0.001		54 (12.6) *p* < 0.001				172 (40.1)	17 (4)
		**Placebo** (*n* = 216)	13 (6)		56 (25.9)		25 (11.6)		8 (3.7)				82 (38)	7 (3.2)
	**Cohort 2** ***n* = 367** (open-label)												146 (39.8)	23 (6.3)
	**Mantainance** (52 weeks) ***n* = 457 patients** (responsible to ozanimod during the induction)	**Ozanimod 1 mg once daily** (*n* = 230)	85 (37) *p* < 0.001		138 (60) *p* < 0.001		105 (45.7) *p* < 0.001		68 (29.6) *p* < 0.001	41 (17.8) *p* = 0.003	73 (31.7) *p* < 0.001	41 (52) *p* = 0.002	113 (49.1)	12 (5.2)
		**Placebo** (*n* = 227)	42 (18.5)		93 (41)		60 (26.4)		32 (14.1)	22 (9.7)	38 (16.7)	22 (29)	83 (36.6)	18 (7.9)

**Table 2:** Summary of efficacy and safety of Etrasimod for Ulcerative Colitis. AEs: Adverse Events. SAEs: Serious Adverse Events. MCS: Modified Mayo Clinic Score. LSM: Least Squares Mean. SE: Standard Error.

Trial	Intervention	Primary Endpoint		Secondary Endpoints						Adverse Events	
**Elevate 52** Phase III (*n* = 433) Randomized, double-blind		**Clinical remission** ***n* (%)**	**Clinical remission** ***n* (%)**	**Endoscopic improvement** ***n* (%)**		**Symptomatic remission** ***n* (%)**		**Endoscopic improvement-histological remission** ***n* (%)**		**TOTAL AEs** ***n* (%)**	**SAEs leading to discontinuation** ***n* (%)**
		**Week 12**	**Week 52**	**Week 12**	**Week 52**	**Week 12**	**Week 52**	**Week 12**	**Week 52**		
	**Etrasimod 2 gr** (*n* = 274)	74 (27%) *p* < 0.0001	88 (32%) *p* < 0.0001	96 [35%] *p* < 0.0001	94 (33%) *p* < 0.0001	126 [46%] *p* < 0.0001	113 (39%) *p* < 0.0001	58 [21%] *p* < 0.0001	127 (44%) *p* < 0.0001	260 (71)	20 (7)
	**Placebo ** (*n* = 135)	10 (7%)	9 (7%)	19 [14%]	11 (8%)	29 [21%]	19 (13%)	6 [4%]	28 (19%)	81 (56)	9 (6)
**Elevate 12** phase III (*n* = 354) Randomized, double-blind		**Clinical remission week 12** ***n* (%)**		**Endoscopic improvement** ***n* (%)**		**Symptomatic remission** ***n* (%)**		**Endoscopic improvement-histological remission** ***n* (%)**		**Total AEs** ***n* (%)**	**SAEs leading to discontinuation** ***n* (%)**
	**Etrasimod 2 gr** (*n* = 222)	55 (25%) *p* = 0.026		68 [31%] *p* = 0.0092		104 [47%] *p* = 0.0013		36 [16%] *p* = 0.036		112 (47)	6 (3)
	**Placebo** (*n* = 112)	17 (15%) *p* = 0.026		21 [19%]		33 [29%]		10 [9%]		54 (47)	2 (2)
**OASIS ** Phase II (*n* = 156) Randomized, double-blind		**Improvement from** **baseline in the modified MCS at week 12** **LSM (SE)**		**Endoscopic improvement** **(%)**		**Improvement in the 2-component MCS** **LSM (SE)**		**Improvement in Total MSC** **LSM (SE)**		**Total AEs** ***n* (%)**	**SAEs leading to discontinuation** ***n* (%)**
	**Etrasimod 1 gr** (*n* = 50)	1.94 (0.31) *p* = 0.146		22.5 *p* = 0.306		1.30 (0.22) *p* = 0.086		2.69 (0.41) *p* = 0.128		31 (59.6)	3 (5.8)
	**Etrasimod 2 gr** (*n* = 52)	2.49 (0.31) *p* = 0.009		41.8 *p* = 0.003		1.75 (0.22) *p* = 0.002		3.35 (0.41) *p* = 0.010		28 (56.0)	4 (8.0)
	**Placebo** (*n* =54)	1.50 (0.30)		17.8		0.92 (0.21)		2.08 (0.39)		27 (50.0)	0

## Data Availability

No new data were generated or analyzed in support of this research.
